# Applying Dynamic Priority Scheduling Scheme to Static Systems of Pinwheel Task Model in Power-Aware Scheduling

**DOI:** 10.1155/2014/587321

**Published:** 2014-07-08

**Authors:** Ye-In Seol, Young-Kuk Kim

**Affiliations:** ^1^Green Energy Institute, Sangmyung University, Seoul 110-743, Republic of Korea; ^2^Deptartment of Computer Science & Engineering, Chungnam Nat'l University, Daejeon 305-764, Republic of Korea

## Abstract

Power-aware scheduling reduces CPU energy consumption in hard real-time systems through dynamic voltage scaling (DVS). In this paper, we deal with pinwheel task model which is known as static and predictable task model and could be applied to various embedded or ubiquitous systems. In pinwheel task model, each task's priority is static and its execution sequence could be predetermined. There have been many static approaches to power-aware scheduling in pinwheel task model. But, in this paper, we will show that the dynamic priority scheduling results in power-aware scheduling could be applied to pinwheel task model. This method is more effective than adopting the previous static priority scheduling methods in saving energy consumption and, for the system being still static, it is more tractable and applicable to small sized embedded or ubiquitous computing. Also, we introduce a novel power-aware scheduling algorithm which exploits all slacks under preemptive earliest-deadline first scheduling which is optimal in uniprocessor system. The dynamic priority method presented in this paper could be applied directly to static systems of pinwheel task model. The simulation results show that the proposed algorithm with the algorithmic complexity of *O*(*n*) reduces the energy consumption by 10–80% over the existing algorithms.

## 1. Introduction

Energy consumption issues are becoming more important for mobile or battery-operated systems. These mobile or portable embedded systems could be stand-alone or wireless networked. In the case of wireless networked system, much research effort was done on managing energy consumption of overall network [1, 2] or radio components [3]. On the other hand, energy consumption issues in the stand-alone embedded system or individual processing component in networked system have another side of problem because the applications in these systems have real-time requirements, so we should guarantee the applications from missing deadlines while reducing energy consumption.

Since the energy consumption of CMOS circuits, adopted in various microprocessors which are generally used in mobile or portable systems, has a quadratic dependency on the operating voltage (*E* *α* *V* ^2^) [4], it is a very useful method for reducing energy consumption to lower the operating voltage of circuits. But, lowering the operating voltage also decreases its clock speed, so, the execution times of tasks are prolonged. This makes the problem more complex for embedded hard real-time systems, where timing constraints of tasks should be met. There has been significant research effort on dynamic voltage scaling (DVS) for real-time systems to reduce energy consumption while satisfying the timing constraints [5–12].

DVS algorithm depends on scheduling policy, task model, and processor architecture. Scheduling policy depends on the priorities of tasks which could be static or dynamic. Generally, static priority system is more manageable in embedded or ubiquitous systems, but more complicated or limited in the aspect of power-saving [13]. One of the static systems is the system of pinwheel task model and its system behavior is really static and predetermined in the sense of online task execution. So, there have been many static approaches to power-aware scheduling in pinwheel task model. But we will show that pinwheel task model could adopt the power-aware scheduling results of dynamic priority systems, so, it is more simple and effective in power-saving.

Among the dynamic priority scheduling policies, we consider earliest-deadline-first (EDF) scheduling policy [14] to be applied to pinwheel task model. EDF is known as optimal in uniprocessor system and has been applied to various task models, for example, periodic, sporadic, or aperiodic [15] task models. We present an algorithm which adopts and improves the result of CC-EDF [9] which is a power-aware version of EDF. The proposed power-aware scheduling method based on dynamic priority could be applied directly to static systems of pinwheel task model and can acquire more effective power-saving than the previous static schemes. The simulation results show that the proposed algorithm reduces energy consumption by 10–80% over the existing algorithms.

The rest of this paper is organized as follows. In Section 2, we present the system model and notations adopted in this paper and introduce the previous work which motivates the work done in this paper. In Section 3, we introduce an approach based on dynamic priority to the pinwheel task scheduling and present a power-aware scheduling algorithm which is applied directly to pinwheel task model. In Section 4, simulation results will be provided and Section 5 will conclude and discuss the future directions of this paper.

## 2. Motivation

In this section, we present the system model and introduce the result of related work.

### 2.1. System Model

Pinwheel task model was introduced in [16] to schedule distance-constrained real-time tasks. This kind of tasks should be executed in distance-constrained manner; that is, the distance of two consecutive jobs of a task, which is defined as the difference of their finishing times, is constrained.

In a distance-constrained task set *T*_*c*_ = {*T*_1_, *T*_2_,…, *T*_*n*_}, every task *T*_*i*_ consists of infinite sequence of jobs *J*_*i*,1_, *J*_*i*,2_,…. Let *f*_*i*,*j*_ denote the finish time of job *J*_*i*,*j*_, for 1 ≤ *i* ≤ *n* and *j* ≥ 1. Then, the distance between two consecutive jobs of a task *T*_*i*_ is limited by *c*_*i*_; that is, *f*_*i*,*j*+1_ − *f*_*i*,*j*_ ≤ *c*_*i*_. Also, there is a precedence constraint between consecutive jobs; that is, *J*_*i*,*j*+1_ can be started only after job *J*_*i*,*j*_ has been finished, and we assume that each job request is ready to be executed as soon as its precedent job is finished. Moreover, jobs are preemptable.

Based on the work done in [16, 17], pinwheel task model was applied to schedule successfully distance-constrained task set. By transforming distance-constrained task sets using pinwheel model, each task is converted to normal periodic task and periods of all tasks are harmonic [16, 17]. Then, rate-monotonic (RM) [14] scheduling policy of static priority systems could be applied.

As stated in [18], video systems need to ensure the interarrival time between frame relays is constrained rather than being just periodic. Also, some applications could not accept pinwheel transformation, because transformed period is shorter than original one. In this case, we may make a job of a task idle in order to prevent it from being executed too frequently [18].

Now, we can introduce periodic or sporadic task system because pinwheel task model could be considered as a special form of periodic task system. Each task of the system we considered is mutually independent. The target processor is DVS enabled uniprocessor and its supply voltage and frequency are varied continuously between [*v*_min⁡_, *v*_max⁡_] and [*f*_min⁡_, *f*_max⁡_], respectively. Let *T* = {*T*_1_, *T*_2_,…, *T*_*n*_} be a set of periodic or sporadic tasks. Each task is represented as *T*_*i*_ = (*P*_*i*_, *C*_*i*_, *D*_*i*_), where

- *P*_*i*_ is period for periodic task, minimum interarrival time for sporadic task, or distance constraint of pinwheel task;
- *C*_*i*_ is the worst-case computation time for a task *T*_*i*_ at the maximum frequency;
- *D*_*i*_ is relative deadline of a task *T*_*i*_.

If an instance or job of a task *T*_*i*_ is released at *R*_*i*_, then its absolute deadline (*d*_*i*_) is *R*_*i*_ + *D*_*i*_. We will consider only tasks with *D*_*i*_ = *P*_*i*_, so, a task *T*_*i*_ could be represented as (*P*_*i*_, *C*_*i*_). Also, the following notations will be used.

- TU_*i*_: the worst-case utilization of a task *T*_*i*_ at the maximum frequency; that is, TU_*i*_ = *C*_*i*_/*P*_*i*_.
- TU: total utilization of all tasks in the system; that is, TU = ∑_*i*_TU_*i*_.
- CC_*i*_: a task's actual computation time which should be less than *C*_*i*_.
- CU_*i*_: actual utilization of a task; that is, CU_*i*_ = CC_*i*_/*P*_*i*_.
- CU: actual total utilization of the system; that is, CU = ∑_*i*_CU_*i*_.
- RC_*i*_: a task's remaining computation time.
- *α*: current frequency ratio, that is, *f*_cur_/*f*_max⁡_.
- TU^*α*^: total utilization of all tasks when processor speed is *α*.

### 2.2. Related Work

The previous work on power-aware scheduling for pinwheel task model is based on offline algorithm [19], which could not utilize slacks of early completed tasks during rum-time, or based on online algorithms which require high order of algorithmic complexity [18, 20] or deal with static priority tasks [21]. Our algorithm is based on dynamic priority scheduling scheme in spite of the fact that pinwheel task system is static one.

EDF, an optimal dynamic priority scheduling policy, has been extensively investigated in the area of real-time and power-aware scheduling [7–11, 14]. While devising a new power-aware scheduling algorithm, we especially considered the result presented by Pillai and Shin [9]. They introduced a cycle-conserving method to real-time DVS. This method reduces the operating frequency on each task completion and increases on each task release. When a task completes its current invocation after using CC_*i*_ computation time, they treat the task as if its worst-case execution time was CC_*i*_. So, processor speed could be set as the actual total utilization CU which is always less than or equal to the worst-case total utilization TU.

**Figure 1:**
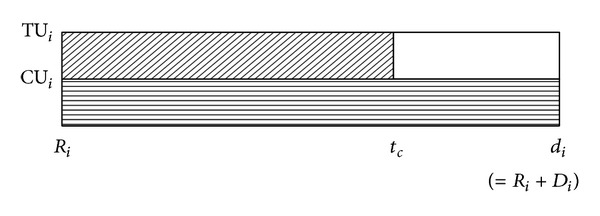
CC-EDF schedule and its available slack.

Mei et al. [8] integrated the above cycle-conserving method and the result of Qadi et al. [10] for sporadic task set. But these methods do not fully utilize the slacks generated. Let us see Figure 1. If a task is complete at *t*_*c*_, then the system has operated at higher frequency than required during the time interval [*R*_*i*_, *t*_*c*_]. This observation provides a clue to slow down the processor speed more when a task is complete. We will show later that the number of slacks which could be used for lowering processor frequency is related to temporal idleness of the completed task.

## 3. Dynamic Priority Scheme for Pinwheel Task Model

### 3.1. Behavior of Pinwheel Task Model

In this section, we investigate the property of pinwheel task model and its behavior of scheduling result. As stated in Section 2, pinwheel task model was introduced to schedule distance-constrained task set.

Let *T*_*c*_ = {*T*_1_, *T*_2_,…, *T*_*n*_} be a distance-constrained task set and let {*c*_1_, *c*_2_,…, *c*_*n*_} be the distance constraint of each task *T*_*i*_, respectively. Without loss of generality, we can assume *c*_1_ ≤ *c*_2_ ≤ ⋯≤*c*_*n*_. Then, using algorithm Sr [16], the original distance constraints are transformed to harmonic ones; that is, each *c*_*i*_ is 2^*n*^ multiples of one number Sr. This transformation reduces the distance-constrained scheduling problem into normal periodic task execution, where each periodic task's period is the same as the transformed distance constraint of corresponding distance-constrained task.

**Figure 2:**
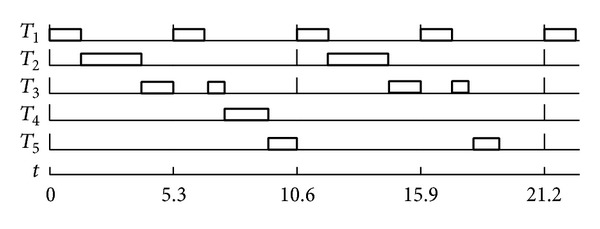
A scheduling result of pinwheel task model.

Suppose a distance-constrained task system consists of five tasks with distance constraints {9.2, 10.6, 10.8, 21.2, 22.6} and their worst-case computation times are {1.5, 2.9, 1.7, 2.1, 1.7}, respectively. After pinwheel transformation [16], the five tasks will have distance constraints {5.3, 10.6, 10.6, 21.2, 21.2}. If we compose a periodic task set where each task's period is distance constraint of original task, optimal static priority scheduling policy like rate-monotonic scheduler [14] could be used to satisfy the distance constraints.  Figure 2 shows the execution sequences generated by rate-monotonic scheduling for the above example when each task executes at its worst-case computation time. As you can see at this figure, the execution schedule for each task has no jitter; that is, each task's relative starting and ending times are fixed. So, the pinwheel task scheduling is more predictable compared to normal periodic task system with fixed priority.

It is known that if total utilization of a task system after pinwheel transformation is lower than or equal to 1, then the task system is schedulable [16]. So, the following theorem holds immediately.

**Theorem 1 .**

If *TU* of a pinwheel task system is *α*, one can schedule it with processor speed *α*.

**Proof**

If we execute the same task at processor speed *α* instead of 1, each task's computation time is *C*_*i*_/*α*. So,

(1) $$\begin{array}{c} {\text{TU}}^{\alpha }=\sum \frac{\left(C_{i}/\alpha \right)}{P_{i}}=\frac{1}{\alpha }\sum \frac{C_{i}}{P_{i}}=\frac{1}{\alpha }\cdot \alpha =1. \end{array}$$

By Theorem 1, we can always maintain total utilization of all tasks as 100% for the pinwheel task system in power-aware scheduling. This property is really the same as that of EDF scheduling. Now, we say that pinwheel scheduling result generated by static priority of rate-monotonic scheduling policy is the same as that generated by dynamic priority of EDF.

**Theorem 2 .**

There is no difference between the scheduling results of rate-monotonic scheduling and EDF in case of pinwheel task model.

**Proof**

Only at the beginning (or the end) of smallest period can start a pinwheel task and also can exist the deadline of each task in pinwheel task model because periods of all tasks are harmonic.

The priority of rate-monotonic scheduling is determined by the length of task's period and is static. That of EDF scheduling is determined by shorter deadline and is dynamic. Now, consider when a task preempts another task. Both rate-monotonic scheduling and EDF preempt lower priority task when a higher priority task arrives and execute the new arrived higher priority task. But the preemption of pinwheel task system can exist only at the beginning (or the end) of the smallest period because all tasks can start only there. The deadline of the higher priority task of rate-monotonic scheduling is always the same as or shorter than that of the preempted lower priority task because periods are harmonic. And if two tasks have the same deadline, let the task of smaller index have higher priority. So, if we index each task by its increasing period at EDF, the preempting higher priority task in rate-monotonic scheduling has also higher priority in EDF than the preempted lower priority task in the case of the same deadline.

For the cases of choosing the highest priority ready task when multiple tasks start at the same time and when the previous task ends or an existing ready task resumes, the selected highest priority task of rate-monotonic scheduling has the shortest deadline and the smallest index because all periods are harmonic. So, all tasks have the same priority orders at every scheduling point between RM and EDF in case of pinwheel task model.

Theorem 2 implies that EDF scheduling system which indexes each task by ascending order of its period and chooses the task with the smallest index when two or more tasks have the same deadline is exactly the same as RM scheduling system for pinwheel task model. So, we can safely apply the result of EDF scheduling policy to pinwheel task system if we impose above two conditions. For the rest of this paper, we assume that EDF scheduling system satisfies them, but this assumption does not sacrifice generality because many or most of EDF systems adopt them.

### 3.2. Power-Aware Scheduling Algorithm

In this section, we introduce a new power-aware scheduling algorithm which adopts dynamic priority scheduling policy. But we already stated in Section 3.1 that the proposed algorithm could be applied to pinwheel task model without modification. Also, it is more effective than the previous work based on EDF as follows.

#### 3.2.1. An Online Algorithm

As we stated in Section 2, the previous algorithms such as CC-EDF [9], DVSST [10], and CC-DVSST [8] do not fully utilize the slacks generated by early completed tasks. Before presenting more discussion, let us introduce a new definition.

**Definition 3 .**

Temporal idleness TI_*i*_(*t*) of a task *T*_*i*_ at time *t* is defined as follows:

(2) 0  until  its  completion  and  after  its  deadline,RCidi−tc  if  it  was  completed  at  time  tc  and  t=tc,TIi(tc)±γ if  t>tc.

The real value of *γ* depends on the status of system and how to calculate it will be presented later. The following lemma computes the available slack at Figure 1 of CC-EDF and CC-DVSST algorithms.

**Lemma 4 .**

At CC-EDF or CC-DVSST scheduling, the amount of computation time exceeding its actual pace until its completion time ( = *t*_*c*_) is the same as [*TI*_*i*_(*t*_*c*_)−(*TU*_*i*_ − *CU*_*i*_)]×(*d*_*i*_ − *t*_*c*_).

**Proof**

Consider

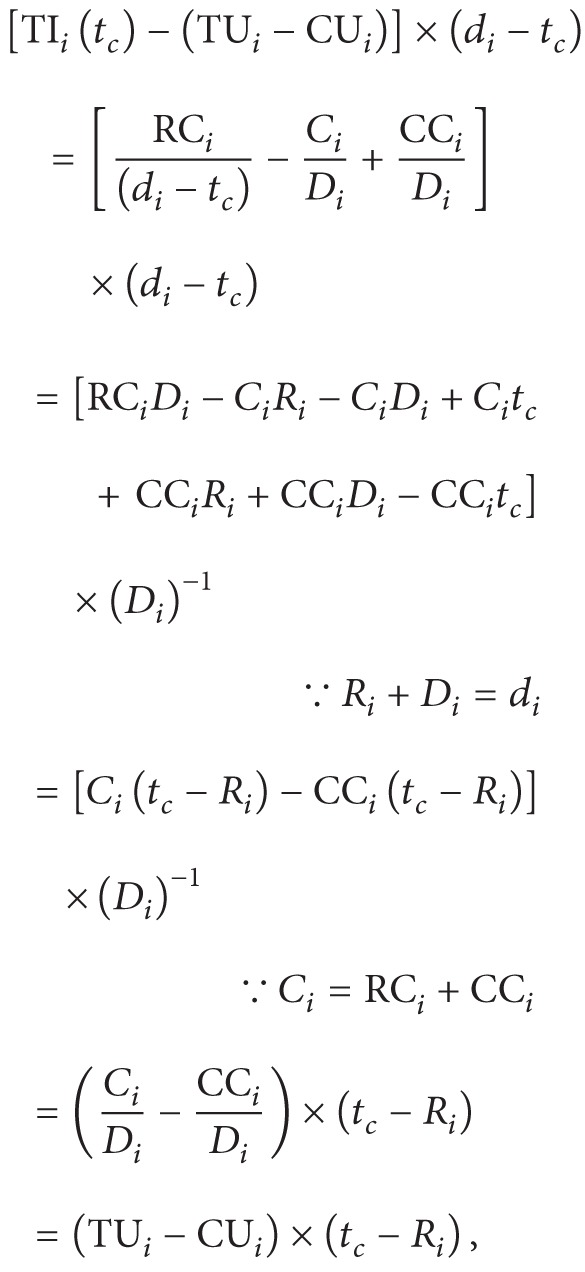
(3)
where “(TU_*i*_ − CU_*i*_)” is the height of the shaded square of Figure 1 and “(*t*_*c*_ − *R*_*i*_)” is the length of its base line, so, “(TU_*i*_ − CU_*i*_) × (*t*_*c*_ − *R*_*i*_)” equals exactly the area of the square and is the same as the amount of computation time exceeding its actual pace ( = CU_*i*_ × (*t*_*c*_ − *R*_*i*_)).

Using Lemma 4, if a task *T*_*i*_ was complete at time *t*, then we can slow down processing speed by amount of TI_*i*_ when executing lower priority tasks than *T*_*i*_ until its deadline, because CC-EDF or CC-DVSST slows down processing speed by the amount of (TU_*i*_ − CU_*i*_). The proposed algorithm tries to use slacks of already completed higher priority tasks which are necessarily generated by assuming the worst-case execution scenarios and applies the result of CC-EDF when the running task's priority is higher than that of already completed tasks. Also, if we could not utilize those slacks by some reasons, that is, when executing higher priority tasks or when slacks are too large to fully utilize, then we evenly distribute those unused slacks until the corresponding deadlines.

One more consideration occurs when there is idle period. Let us consider a periodic task set *A* = {*T*_*i*_ = (*P*_*i*_, *C*_*i*_)∣*T*_1_ = (3, 1), *T*_2_ = (3, 1), *T*_3_ = (6, 2)}. If *T*_1,1_ and *T*_2,1_ are complete at *t* = 1 and *t* = 2, respectively, and *T*_3,1_ is complete early at *t* = 2.5, then TI_3_(2.5) = 1.5/3.5 = 3/7. If we lower the processing speed as much as TI_3_(2.5), then actual processing capacity during *t* = [3, 6) is (1 − 3/7) × 3 = 12/7 which is less than the sum of WCETs of *T*_1,2_ and *T*_2,2_.

**Figure 3:**
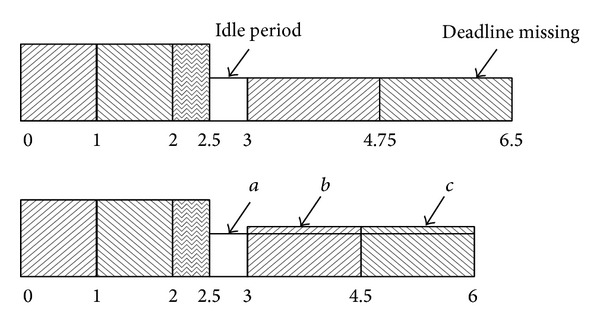
A slack reduction example.

Deadline missing occurs because there is idle period *t* = [2.5, 3). During the idle period, the total processing capacity which should be processed under the actual execution scenarios is larger than the sum of slacks used. So, we should reduce the future slacks to compensate this mismatch.  Figure 3 shows it.

The number of slacks which should be reduced is TU − TI and is the same as (CU − (TI − (TU − CU))). In Figure 3, the area “*a*” is the same as “*b*” + “*c*” and if TI exceeds TU, we could save some slacks because only “TU × (length  of  idle  period)” should be processed by CPU.

Now, we will show briefly that the presented algorithm is correct. Theoretic full proof using temporal workload analysis could be found at our previous paper [22].

**Theorem 5 .**

The algorithm presented, in Algorithm 1, schedules every periodic or sporadic task set using EDF if and only if ∑_*i*_ *C*_*i*_/*P*_*i*_ ≤ 1.

**Proof**

“Only If” part: if *U* > 1, then EDF will not find a feasible schedule; therefore, our algorithm will not also find a feasible schedule because our algorithm is the same as EDF or DVSST when tasks execute always at their worst-case execution scenarios; that is, TI_*i*_(*t*) = 0 and TU_*i*_ = CU_*i*_ for ∀*t* and *i*.

“If” part: we will show that total amounts of executed computations using our algorithm are always larger than or equal to those of another ideal system which schedules always tasks without violating their timing constraints. Also, we will show that our algorithm executes jobs of higher priority tasks more than or the same as the ideal system, so, our scheduling algorithm also satisfies the real-time constraints.

**Figure 4:**
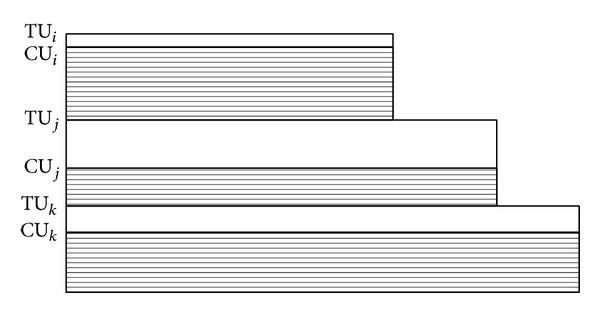
Ideal CPU and ideal execution.

Let us consider ideal CPU of ideal system which can anticipate each task's actual computation time when it is released and can execute active tasks concurrently with the speed of CU. This ideal system of Figure 4 always satisfies all timing constraints and is really the most effective in power consumption.

**Figure 5:**
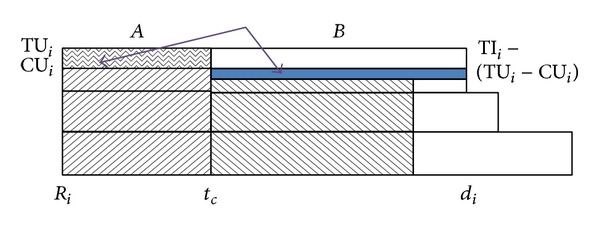
Slack exchange process.

Our real system starts to execute with TU speed and slows down as much as (TU − TI) when a task is completed. And we already said that [TI_*i*_ − (TU_*i*_ − CU_*i*_)] × (*d*_*i*_ − *t*_*c*_) = *B* is exactly the same as (TU_*i*_ − CU_*i*_) × (*t*_*c*_ − *R*_*i*_) = *A*. Therefore, we can apply the substitution operation which replaces some areas of *A* by the same areas of *B* to the scheduling result of our real system.  Figure 5 shows it.

During the time interval [*t*_*c*_, *d*_*i*_] which has no idle period, we can apply the above operation to all areas of *A*. So, total computing capacity of our algorithm is larger than or equal to that of the ideal system of Figure 4. And the computing capacity of the former is always exhausted by tasks which have shorter or the same deadlines of tasks of the latter.

**Figure 6:**
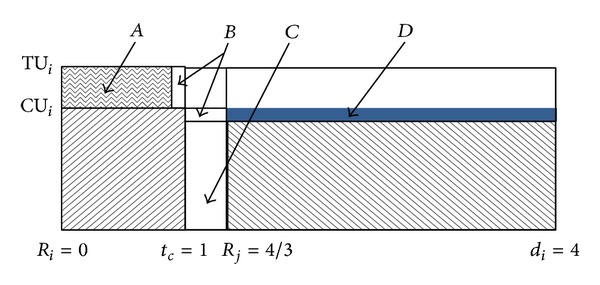
Slack exchange process for idle period.

During idle period, there exist some areas of ideal system which cannot find counterparts of real system. Those areas could be filled up or substituted with the areas of future slacks of already completed tasks as shown in Figure 3 and the above exchange operation could be also applied to them. This substitution consisted of two steps as shown in Figure 6. Area “*D*” (future slack) is substituted by area “*C*” (idle slot) and is exchanged by area “*A*” (a portion of computation time exceeding its actual pace).

Now, total computing capacity of our real system is always larger than or equal to that of the substitution and exchange result. And the computing capacity of the former is always exhausted by tasks which have shorter or the same deadlines of tasks of the latter as we already stated.

#### 3.2.2. An Illustrative Example

**Figure 7:**
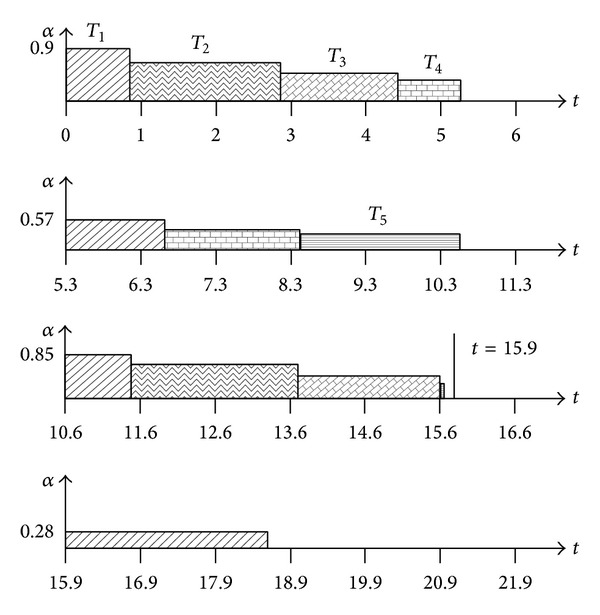
An illustrative example.

Let us consider an illustrative example, a pinwheel task system with distance constraints {9.2, 10.6, 10.8, 21.2, 22.6} and the worst-computation times {1.5, 2.9, 1.7, 2.1, 1.7}, respectively, which is already introduced in Section 3. After pinwheel transformation, distance constraints of the tasks are {5.3, 10.6, 10.6, 21.2, 21.2}, respectively. Now, suppose that actual computation times of the tasks are always {0.75, 1.45, 0.85, 1.05, 0.68}, respectively. Total utilization of the tasks after pinwheel transformation is approximately 0.9.  Figure 7 shows their execution sequences.

At *t* = 0, *T*_1_ starts to execute with processor speed of *α* ≈ 0.9 and, at *t* ≈ 0.84, it is completed. Then, CU_1_ is 0.14 and TI_1_ is 0.17, approximately. *T*_2_ starts now to execute with processor speed of *α* ≈ 0.728  ( = TU − TI_1_ = CU − (TI_1_ − (TU_1_ − CU_1_))) and, at *t* ≈ 2.83, it is completed. Now then, CU_2_ is 0.14 and TI_2_ is 0.19, approximately. The processor speed for *T*_3_ is *α* ≈ 0.54  ( = TU − TI_1_ − TI_2_ = CU − (TI_1_ − (TU_1_ − CU_1_)) − (TI_2_ − (TU_2_ − CU_2_))). *T*_3_ will be completed at *t* ≈ 4.4. *T*_4_ will be executed with processor speed of *α* ≈ 0.4 from *t* ≈ 4.4 to 5.3 and will be preempted by *T*_1_ there. Now, *T*_1_ starts with processor speed of *α* ≈ 0.57  ( = TU − TI_2_ − TI_3_ = CU − (TI_2_ − (TU_2_ − CU_2_)) − (TI_3_ − (TU_3_ − CU_3_))).

Above process will be continued until *t* = 10.6. There, *T*_1_, *T*_2_, and *T*_3_ will be released at the same time and *T*_1_ will preempt *T*_5_. *T*_4_ was already completed at *t* ≈ 8.4 with CU_4_  (≈0.05) and TI_4_  (≈0.082). But *T*_1_ could not use TI_4_ because deadline of *T*_1_ is shorter than that of *T*_4_. So, *T*_1_ will be executed with processor speed of *α* ≈ 0.85 which is the same as CU and it will be completed at *t* ≈ 11.5. During the execution of *T*_1_, *T*_4_'s slack was not used, so, we can increase its temporal idleness TI_4_ into 0.085, approximately. The unused slack is (TI_4_ − (TU_4_ − TI_4_)) × Δ*t* ≈ 0.032 × (11.5 − 10.6) ≈ 0.03 and is distributed evenly until deadline, so, increased ratio is 0.03/(21.2 − 11.5) ≈ 0.003. Now, *T*_2_ will be executed with processor speed of *α* ≈ 0.64  ( = TU − TI_1_ − TI_4_ = CU − (TI_1_ − (TU_1_ − CU_1_)) − (TI_4_ − (TU_4_ − CU_4_))) because deadline of *T*_2_ is the same as that of *T*_4_. *T*_2_ will be completed at *t* ≈ 13.7 and its temporal idleness is 0.195, approximately.

*T*_3_ will be completed at *t* ≈ 15.65. Now, *T*_3_ resumes its execution and will be completed at *t* ≈ 15.69. There is no ready task until *t* = 15.9 when *T*_1_ is released. So, at *t* = 15.9, temporal idleness should be reduced. The last calculated CPU speed is 0.11  ( = TU − ∑*T*_*i*_), and the number of slacks which should be reduced is 0.023  ( = *α* × Δ*t* = 0.11 × (15.9 − 15.69)). This slack is obtained from *T*_2_ until deadline, so, the recalculated TI_2_′ is 0.19  ( = TI_2_ − 0.023/(21.2 − 15.9)), approximately. *T*_1_ will start at *t* = 15.9 with processor speed of *α* ≈ 0.28 and will be completed at *t* ≈ 18.55. Note that the calculated processor speed is exactly “0” at *t* ≈ 18.55.

## 4. Experimental Results

We evaluated our proposed algorithm using RTSIM [23] which is a real-time simulator. RTSIM can simulate the behaviors of dynamic voltage scaling algorithms as well as traditional real-time scheduling algorithms. In this simulation, it is assumed that a constant amount of energy is required for each cycle of operation at a given voltage. This quantum is scaled by the square of the operating voltage, consistent with energy dissipation in CMOS circuits (*E* ∝ *V* ^2^) [4, 7]. Only the energy consumed by CPU was computed and any other sources of energy consumption were ignored. Also, we do not consider preemption overheads, task switch overheads, and operating frequency change overheads. It is also assumed that the CPU consumes no energy during idle period and its operating frequency range is continuous at [*f*_min⁡_ = 0, *f*_max⁡_ = 1].

We compared our proposed algorithm with CC-EDF for periodic task model, with DVSST and CC-DVSST for sporadic task model, and with CC-EDF and CC-RM [9] for pinwheel task model. CC-EDF assumes periodic task model, and CC-DVSST is a direct result of CC-EDF and DVSST. DVSST and CC-DVSST assume sporadic task model, so, we compared them at sporadic task system. CC-RM is a cycle-conserving rate-monotonic scheduling algorithm, so, it adopts static priority task model. CC-RM could be applied to pinwheel task model because of its static property. In spite of the fact that CC-EDF is a dynamic priority scheduling policy, it could be applied to pinwheel task model by Theorem 2. So, we compared our algorithm with CC-RM and CC-EDF at pinwheel task model.

To evaluate the effect of number of tasks in the system, we generated 10 or 20 tasks for each comparison. Their periods or minimum interarrival times are chosen randomly in the interval [1–1000] ms. We divided each task of system into three groups to reflect more real environments. One group of tasks has short period in the interval [1–10] ms, another group of tasks has medium period in the interval [10–100] ms, and the last group of tasks has long period in the interval [100–1000] ms. The simulation was also performed by varying the load ratio of tasks, that is, the ratio of the actual computation time to the worst-case computation time. For the periodic and sporadic task simulations, the worst-case total utilization of system is always 1; that is, TU = 1. For pinwheel task model, the generated task sets were transformed by the single-number reduction technique [17], so, their periods are harmonic and their total utilization after transformation should be less than 1. If the total utilization of a task set after transformation exceeds 1, then it was discarded.

**Figure 8:**
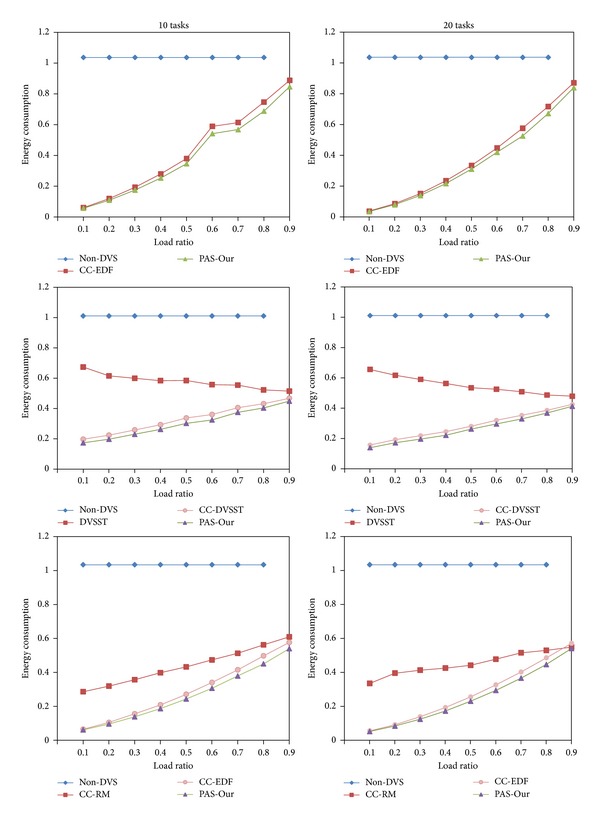
Simulation result for periodic, sporadic, and pinwheel tasks.

Figure 8 shows the simulation result for periodic, sporadic, and pinwheel task systems. For periodic task system, our algorithm always outperforms CC-EDF. For sporadic task system, our proposed algorithm outperforms both DVSST and CC-DVSST. For DVSST, the ratio of energy saving is up to 70% and for CC-DVSST up to 10%. The effect of number of tasks on the system could be also neglected, but the number of CPU frequency changes of our algorithm was larger than that of DVSST and almost the same as that of CC-DVSST. So, the ratio of energy saving to DVSST could be decreased. But our algorithm has huge performance gain to DVSST, so, in spite of frequency change overheads, it is expected that our algorithm still outperforms DVSST at real environments.

For pinwheel task model, our algorithm always outperforms both CC-RM and CC-EDF. CC-EDF always outperforms CC-RM except the case of 20 tasks and 90% load ratio. For the number of CPU frequency changes, that of CC-RM is slightly lesser than those of CC-EDF and our algorithm in the range of small load ratio and almost the same in the range of large load ratio. As you can see, CC-EDF and our algorithm outperform CC-RM by up to 80% in the range of small load ratio, so, we can still insist that dynamic priority scheduling algorithms of CC-EDF and our proposed algorithm have huge performance gain in the power saving in the range of small and medium load ratios.

## 5. Conclusion

In this paper, we investigated the property of pinwheel task model and showed that this task model can adopt the scheduling results of dynamic priority systems. So, this pinwheel task system which is integrating static and dynamic scheduling results is simple enough to be applied to embedded or ubiquitous computing and is more effective in power saving than the systems of only static method. Also, we presented a power-aware scheduling algorithm for general periodic and sporadic task systems and applied it to pinwheel task model without modification. The proposed algorithm adopts the results of cycle conserving method (CC-EDF) and sporadic task scheduling (DVSST) and improves them. The simulation results show that the proposed algorithm outperforms existing algorithms by up to 10–80% with respect to CPU energy saving.

In the future, we would like to improve the proposed algorithm. This could be done if we assign all slacks generated by the early completed higher priority tasks into the task of the highest priority among the uncompleted ready tasks instead of evenly distributing them until the ends of deadlines. This method may lower processor frequency much more than the proposed algorithm. Then, there may be more chances to save power consumption.

## Figures and Tables

**Algorithm 1:**
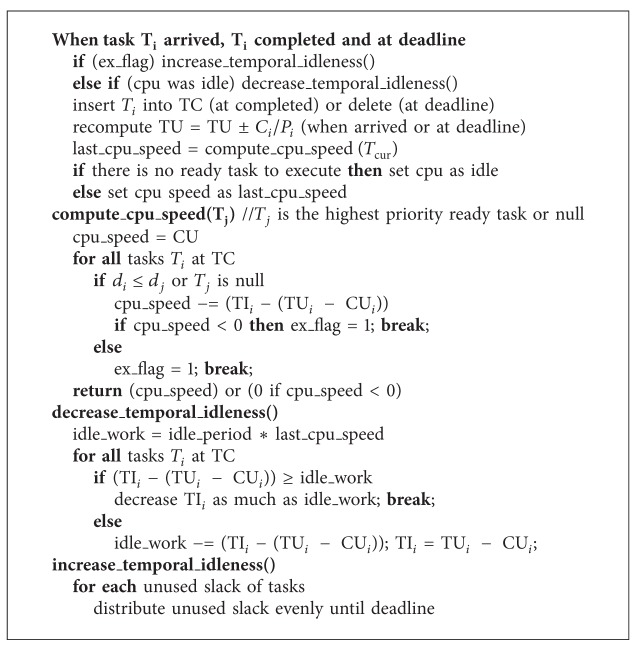
A power-aware  scheduling  algorithm.
